# Supplementary material for: Sulfonated Azocalix[4]arene-Modified Metal–Organic Framework Nanosheets for Doxorubicin Removal from Serum
Source: Nanomaterials (Basel). 2024 May 16;14(10):864. doi: 10.3390/nano14100864 (PMC11124067; doi:10.3390/nano14100864)
Supplement: Supplementary file 1 [file nanomaterials-14-00864-s001.zip › nanomaterials-2992815-supplementary.pdf]

Supplementary Material

Sulfonated Azocalix[4]arene-Modified Metal–Organic Framework Nanosheets for Doxorubicin Removal from Serum

Xiao-Min Cao [1], Yuan-Qiu Cheng [1,2], Meng-Meng Chen [1,2], Shun-Yu Yao [1,2], An-Kang Ying [1,2], Xiu-Zhen Wang [1], Dong-Sheng Guo [1,2,3,*] and Yue Li [1,4,*]

[1] College of Chemistry, Nankai University, Tianjin 300071, China; 2120210908@mail.nankai.edu.cn (X.-M.C.); yqcheng@mail.nankai.edu.cn (Y.-Q.C.); 1120200366@mail.nankai.edu.cn (M.-M.C.); yaosy@mail.nankai.edu.cn (S.-Y.Y.); yak147369@163.com (A.-K.Y.); 2120221095@mail.nankai.edu.cn (X.-Z.W.)

[2] State Key Laboratory of Elemento-Organic Chemistry, Key Laboratory of Functional Polymer Materials (Ministry of Education), Frontiers Science Center for New Organic Matter, Collaborative Innovation Center of Chemical Science and Engineering (Tianjin), Nankai University, Tianjin 300071, China

[3] Xinjiang Key Laboratory of Novel Functional Materials Chemistry, College of Chemistry, and Environmental Sciences, Kashi University, Kashi 844000, China

[4] Key Laboratory of Advanced Energy Materials Chemistry (Ministry of Education), Nankai University, Tianjin 300071, China

* Correspondence: dshguo@nankai.edu.cn (D.-S.G.); liyue84@nankai.edu.cn (Y.L.)

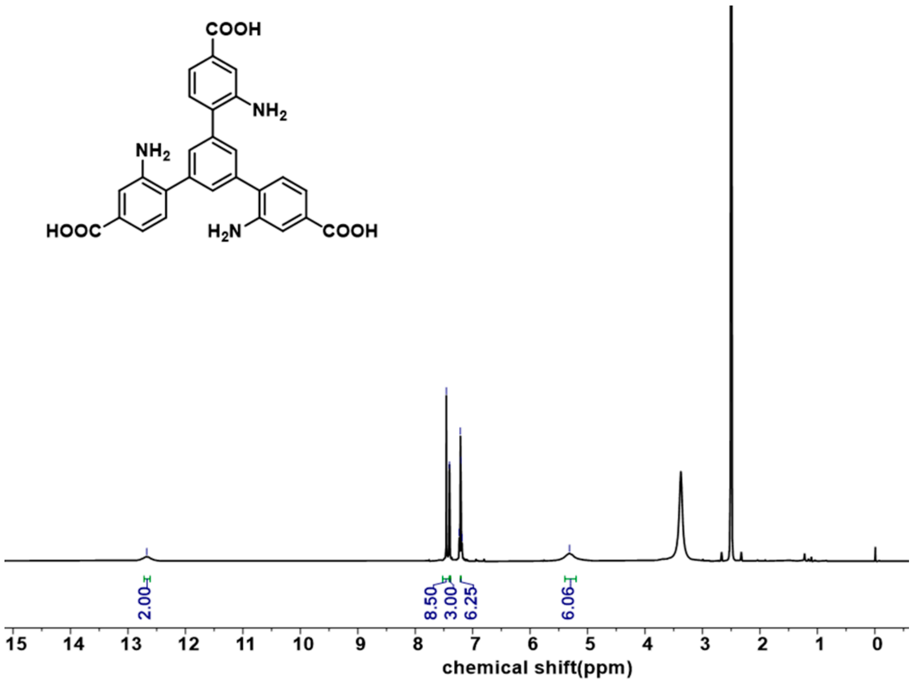

Figure S1. ¹H NMR spectrum of $H_3BTB\text{-}3NH_2$.

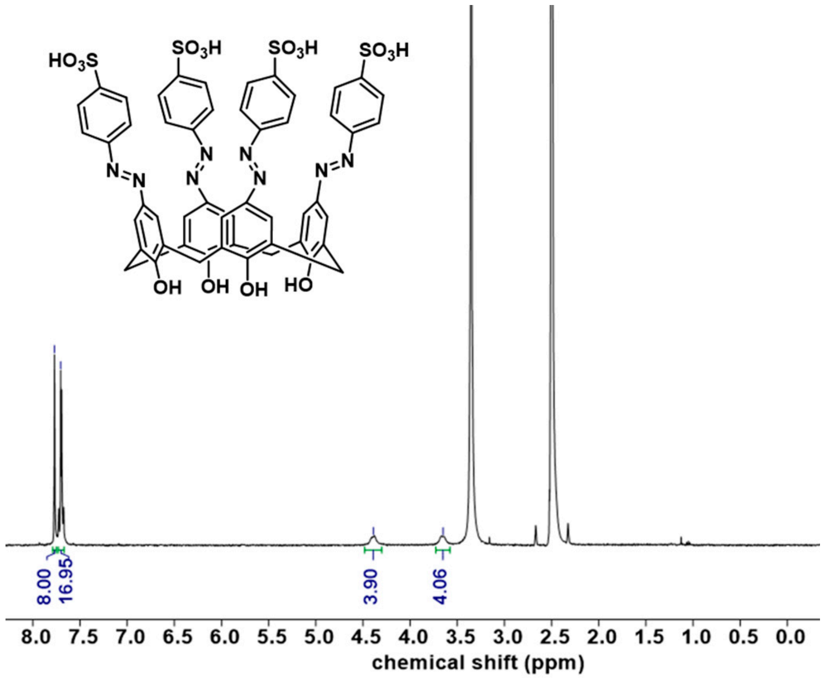

Figure S2. ¹H NMR spectrum of SAC4A.

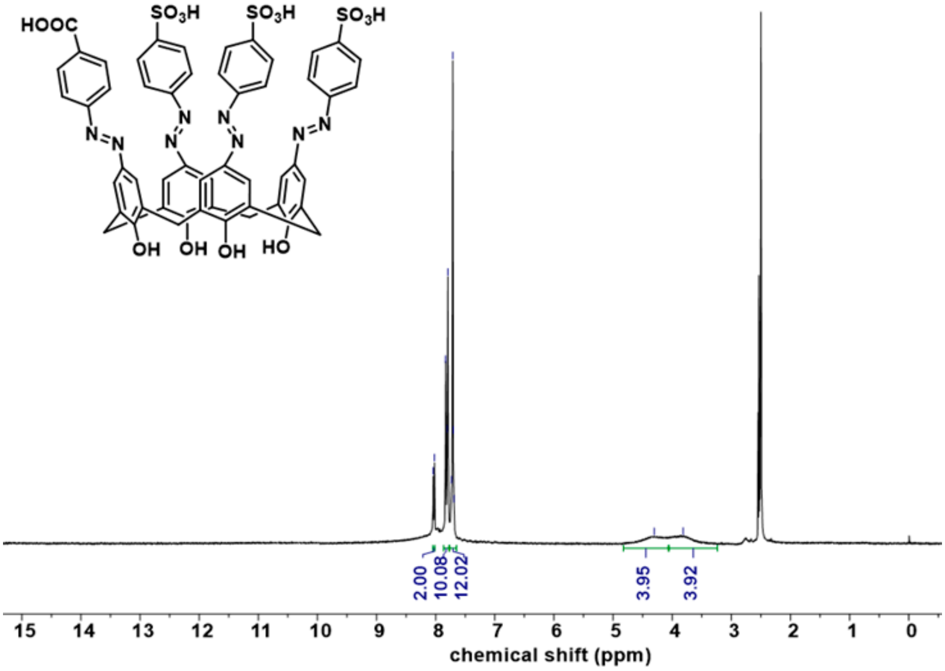

Figure S3. ^{1}H NMR spectrum of CSAC4A.

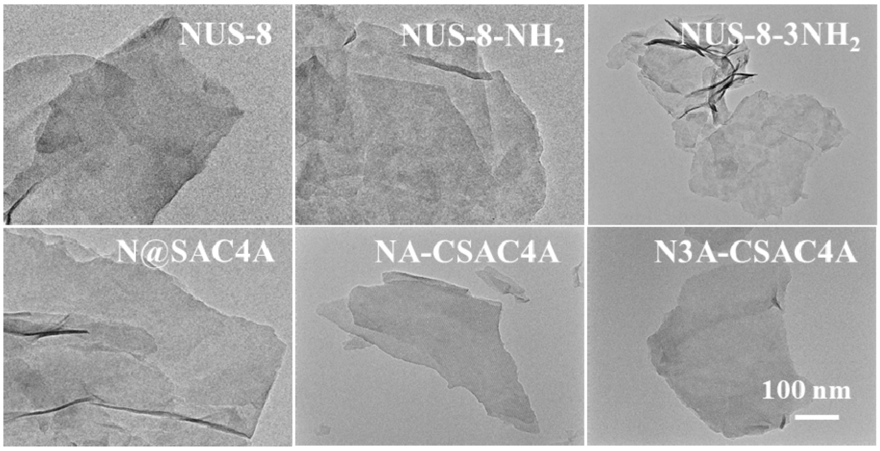

Figure. S4. TEM images of MOF nanosheet precursors and SAC4A/CSAC4A-modified products.

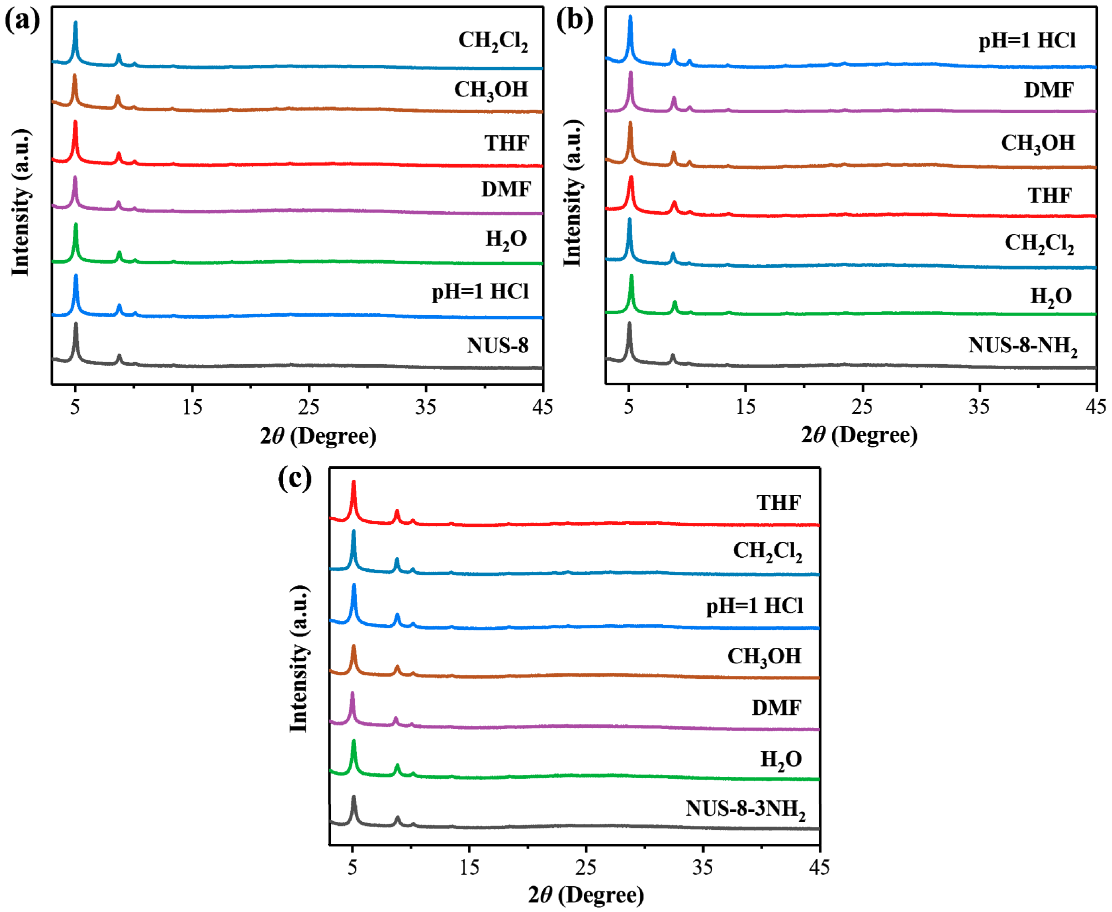

Figure S5. PXRD patterns of the samples of **(a)** NUS-8, **(b)** NUS-8-NH_2 and **(c)** NUS-8-3NH_2 after the immersion in various solvents for 24 h.

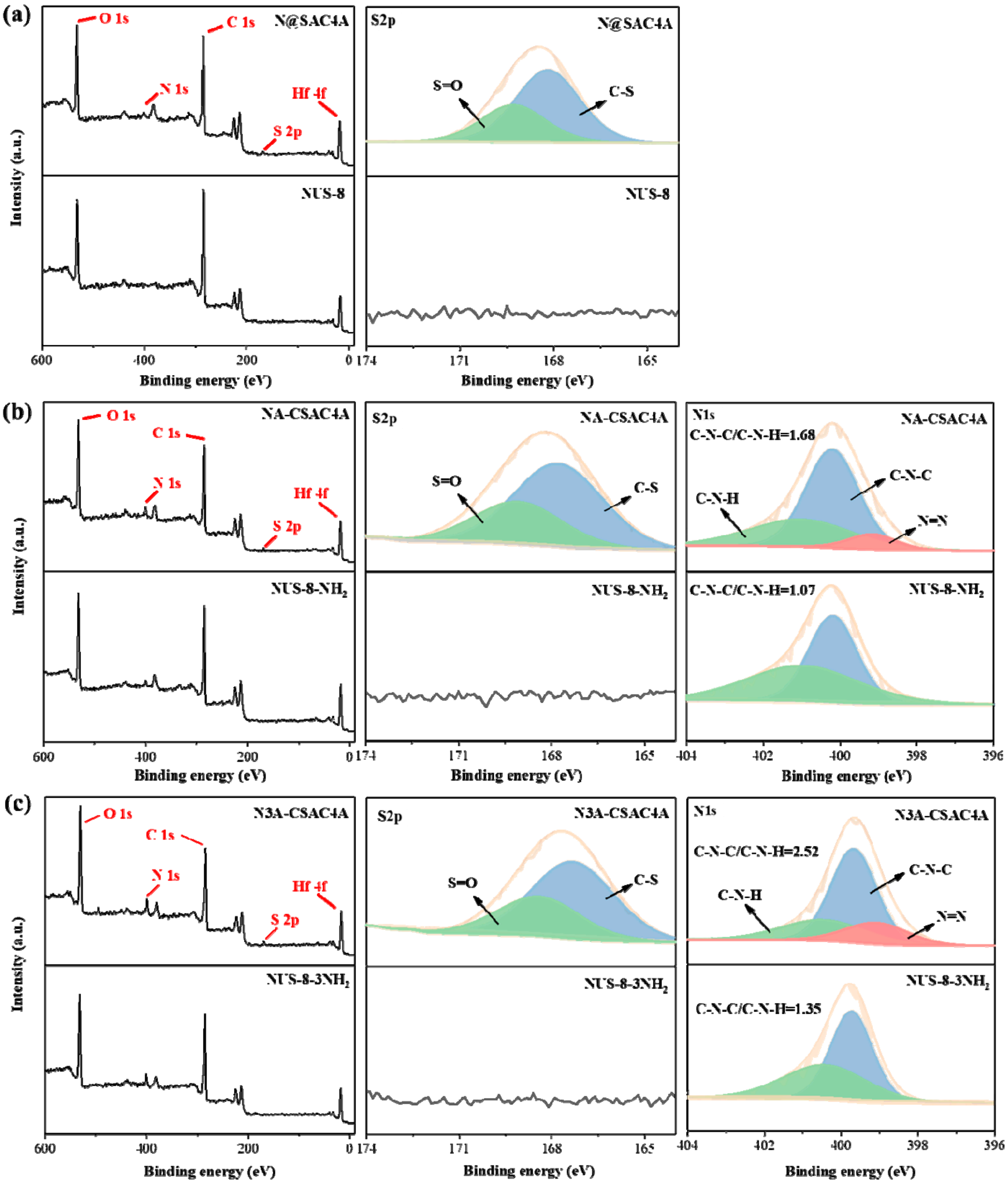

Figure S6. XPS spectra of **(a)** N@SAC4A, **(b)** NA-CSAC4A, **(c)** N3A-CSAC4A and their MOF nanosheet precursors.

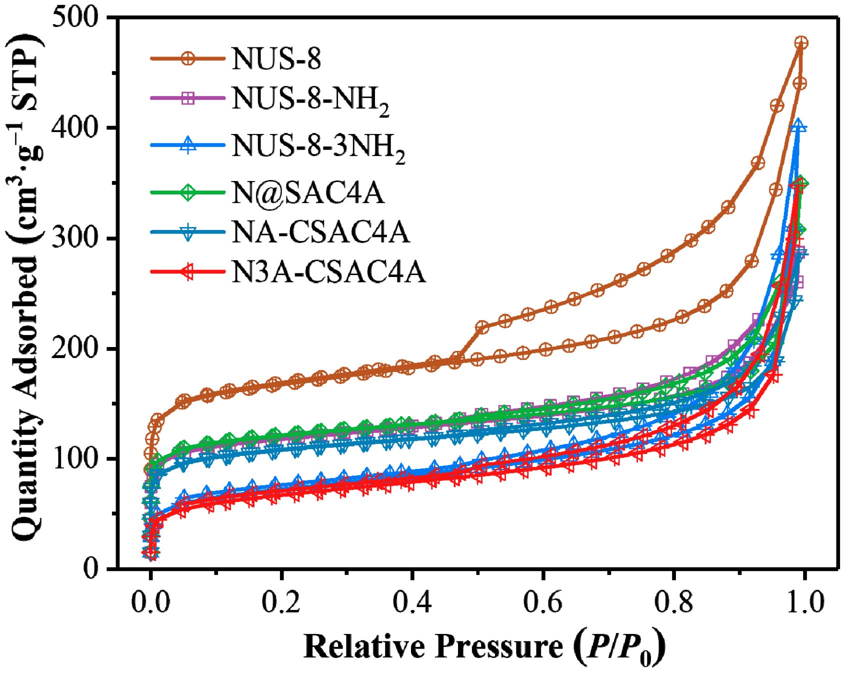

Figure S7. N_2 sorption isotherms of SAC4A/CSAC4A-modified and unmodified MOF nanosheets.

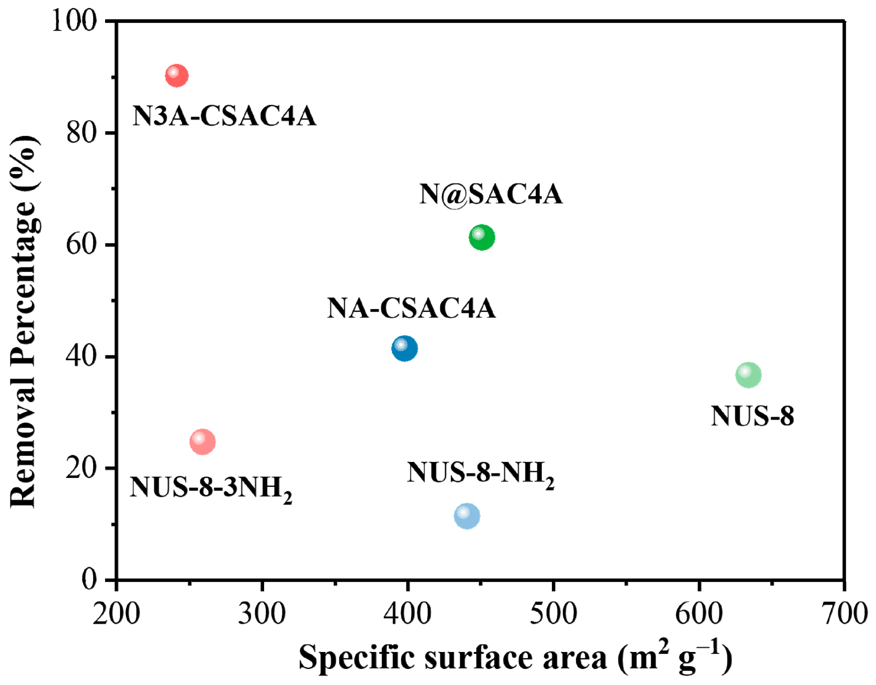

Figure S8. Relevance between the adsorption performance and specific surface area of SAC4A/CSAC4A-modified and unmodified MOF nanosheets.

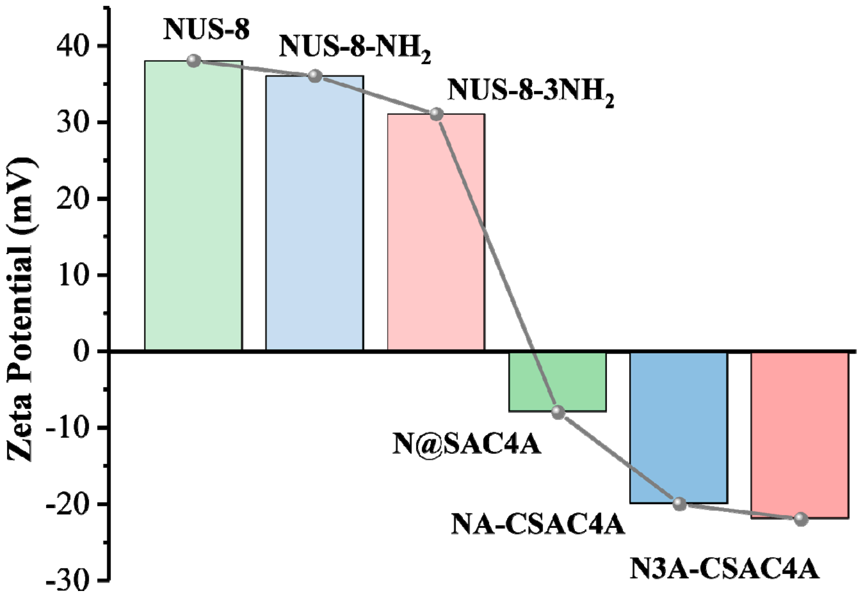

Figure S9. ζ-potentials of N@SAC4A, NA-CSAC4A, N3A-CSAC4A and their MOF nanosheet precursors.

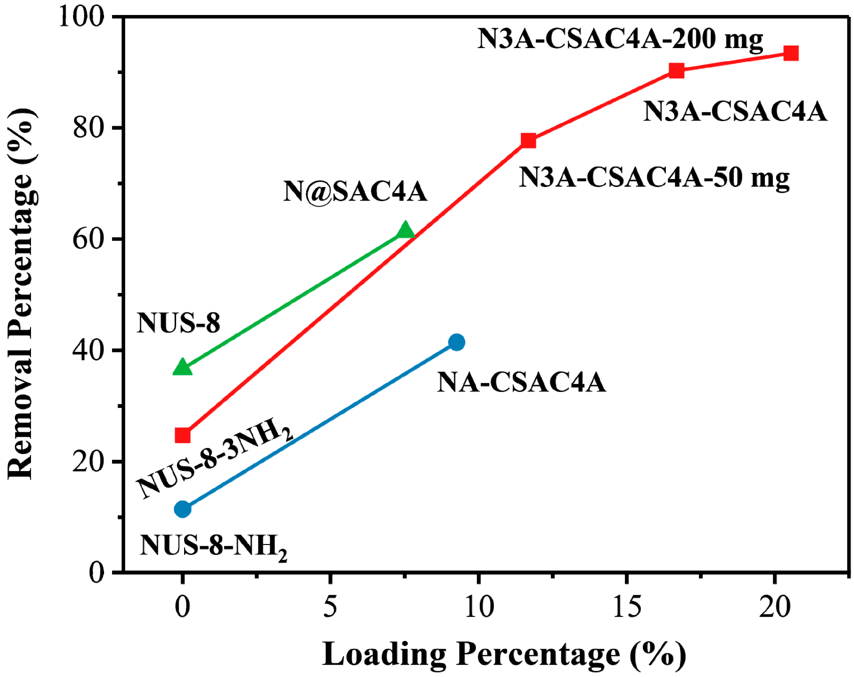

Figure S10. Change of DOX removal percentage with the loading amount of SAC4A/CSAC4A on the adsorbent.

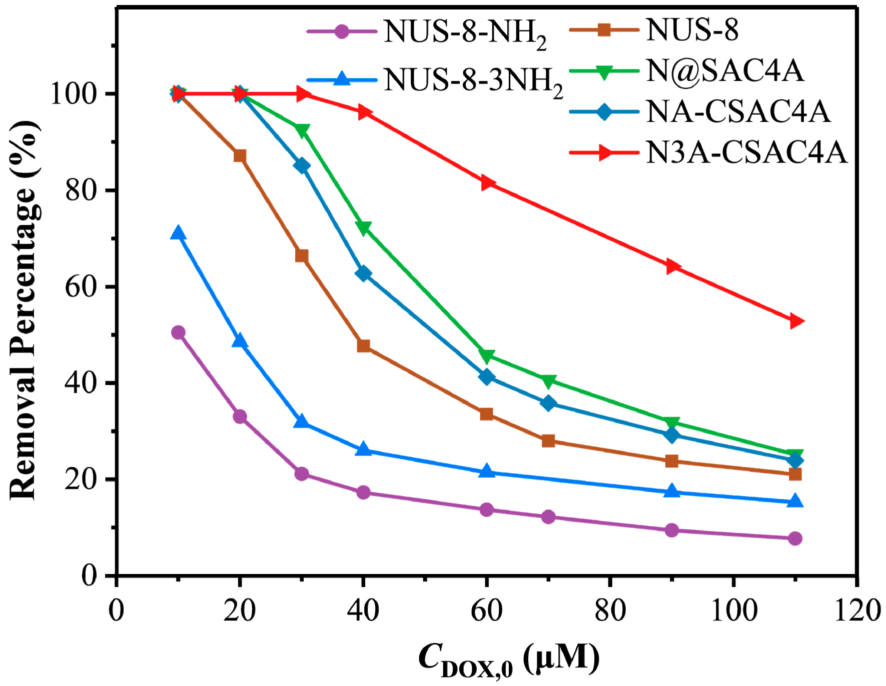

Figure S11. Changes of DOX removal percentage with initial DOX concentration.

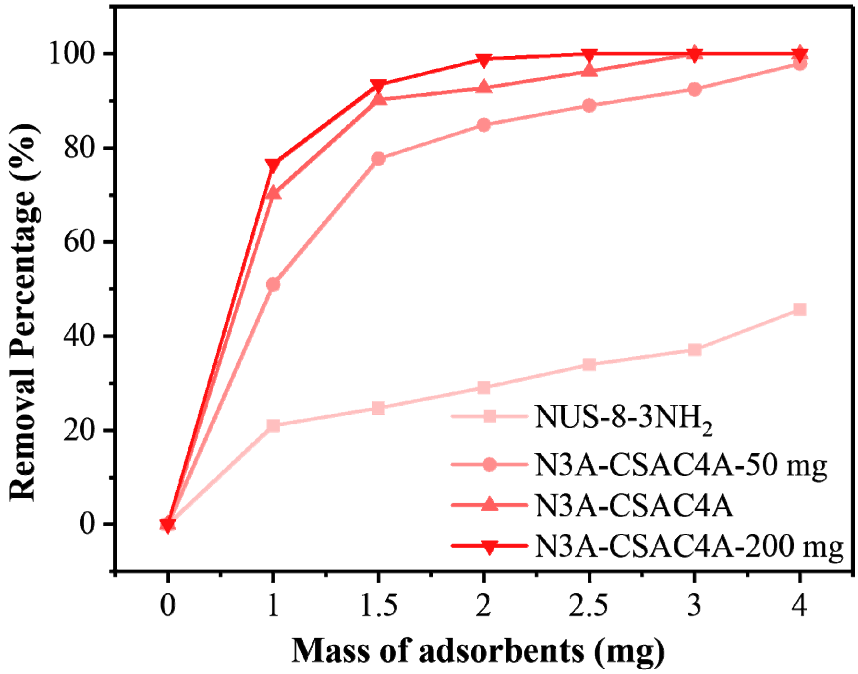

Figure S12. DOX removal by NUS-8-3NH$_2$ and the N3A-CSAC4A samples prepared with different CSAC4A addition amounts, $C_{DOX,0}$ = 50 μM. In the synthesis of N3A-CSAC4A samples, the addition amounts of CSAC4A was changed from 100 mg to 50 and 200 mg, and the addition amounts of DIPEA and HATU were adjusted according to that of CSAC4A.

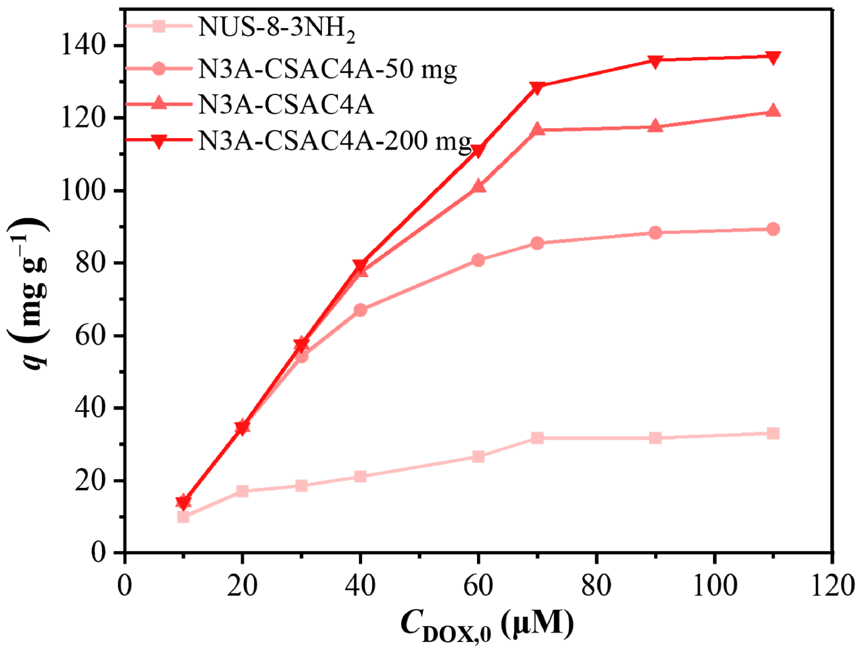

Figure S13. Adsorbed amounts of DOX by NUS-8-3NH2 and the N3A-CSAC4A samples prepared with different CSAC4A addition amounts, 0.3 mg mL^{-1} adsorbent, $C_{DOX,0}$ = 10–110 μM.

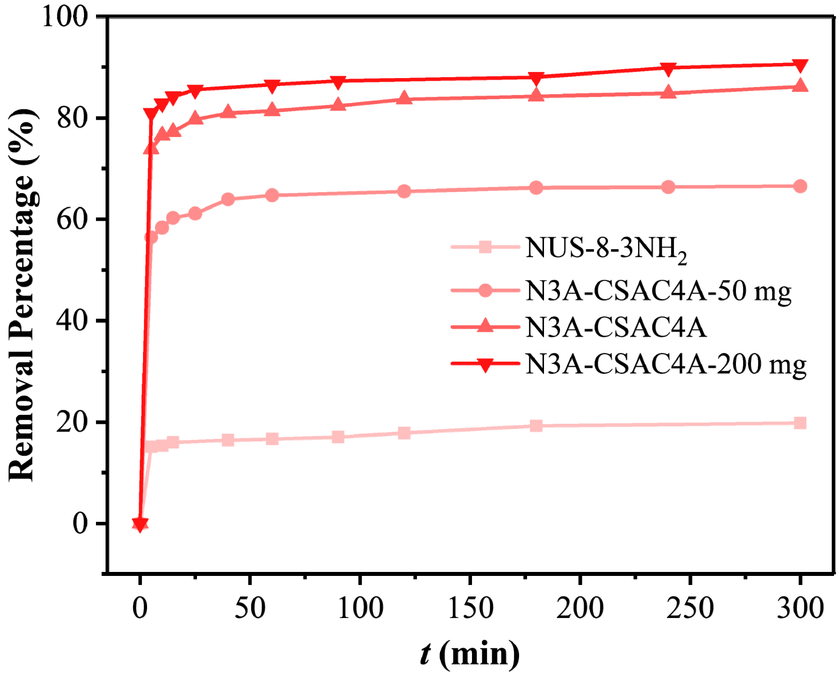

Figure S14. DOX removal percentages at different time intervals by NUS-8-3NH2 and the N3A-CSAC4A samples prepared with different CSAC4A addition amounts.

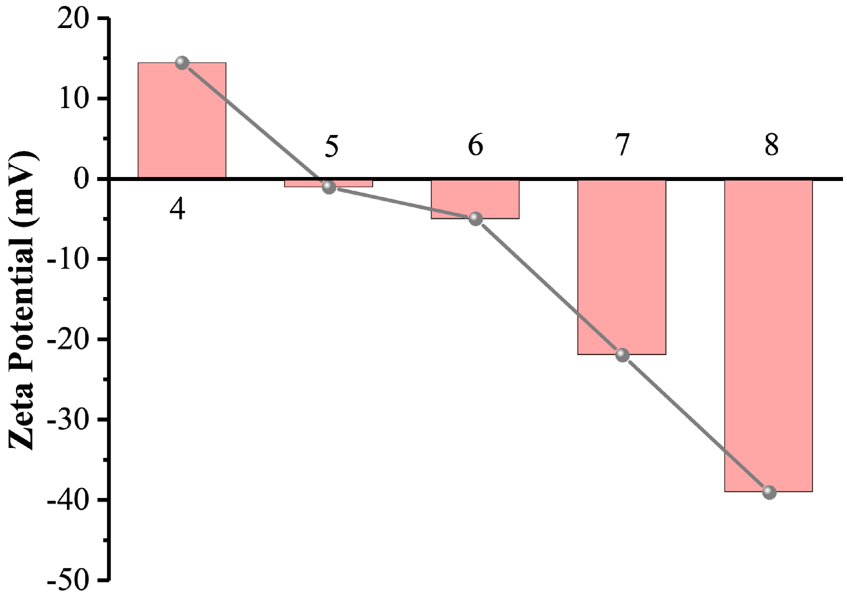

Figure S15. ζ-potentials of N3A-CSAC4A at different pH values.

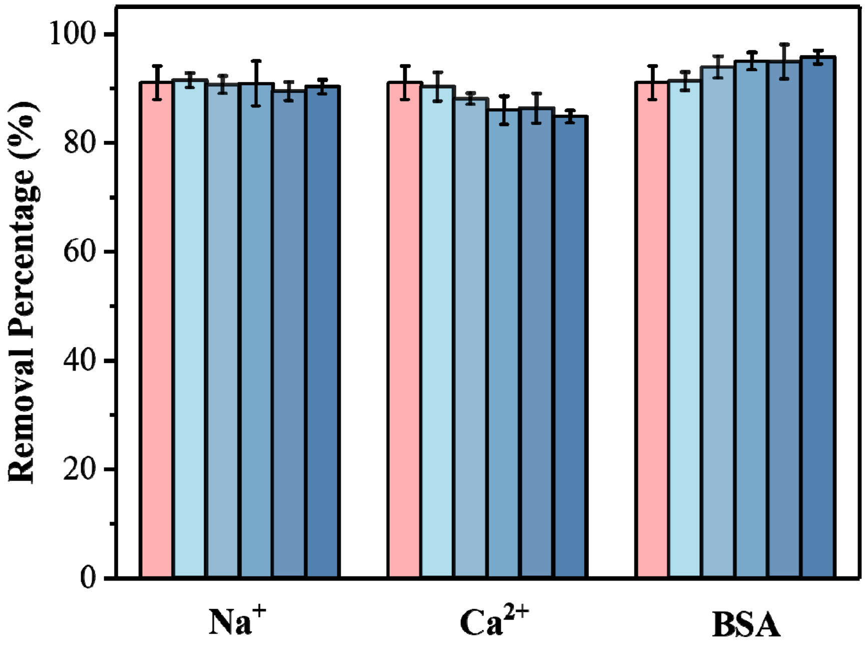

Figure S16. DOX removal by N3A-CSAC4A in the presence of Na^{+} (from left to right: 0, 80, 110, 140, 170 and 190 mM)、Ca^{2+} (from left to right: 0, 3, 30, 50, 70 and 90 mM) and BSA (from left to right: 0, 15, 25, 35, 45 and 55 mM).

Table S1. ICP-OES results and the calculated loading amounts of SAC4A/CSAC4A.

Precursor	Modified product	S (wt. %)	Hf (wt. %)	SAC4A/CSAC4A (wt. %)
NUS-8	N@SAC4A	0.27	36.93	7.53
NUS-8-NH_2	NA-CSAC4A	0.21	33.21	9.26
NUS-8-3NH_2	N3A-CSAC4A	0.29	28.45	16.69
	N3A-CSAC4A-50 mg	0.20	28.46	11.68
	N3A-CSAC4A-200 mg	0.34	27.98	20.55
